# Improving feature selection performance using pairwise pre-evaluation

**DOI:** 10.1186/s12859-016-1178-3

**Published:** 2016-08-20

**Authors:** Songlu Li, Sejong Oh

**Affiliations:** 1 Department of Nanobiomedical Science, Dankook University, Cheonan, 330-714 Korea; 2 Department of Computer Science and Technologies, Yanbian University of Science & Technology, Yanji City, China

**Keywords:** Classification, Feature interaction, Feature selection, Filter method

## Abstract

**Background:**

Biological data such as microarrays contain a huge number of features. Thus, it is necessary to select a small number of novel features to characterize the entire dataset. All combinations of the features subset must be evaluated to produce an ideal feature subset, but this is impossible using currently available computing power. Feature selection or feature subset selection provides a sub-optimal solution within a reasonable amount of time.

**Results:**

In this study, we propose an improved feature selection method that uses information based on all the pairwise evaluations for a given dataset. We modify the original feature selection algorithms to use pre-evaluation information. The pre-evaluation captures the quality and interactions between two features. The feature subset should be improved by using the top ranking pairs for two features in the selection process.

**Conclusions:**

Experimental results demonstrated that the proposed method improved the quality of the feature subset produced by modified feature selection algorithms. The proposed method can be applied to microarray and other high-dimensional data.

## Background

Microarray gene expression data contains thousands of hundreds of genes (features). Biologists are interested in identifying the expressed genes that correlate with a specific disease, or genes with strong interactions. The high dimensionality of microarray data is a challenge for computational analysis. Feature selection by data mining may provide a solution because it can deal with high dimensional datasets [1].

The goal of feature selection is to find the best subset with fewer dimensions, but that also contributes to higher prediction accuracy. This speeds up the execution time for the learning algorithms before data analysis as well as improving the prediction accuracy. A simplistic way of obtaining the optimal subset of features is to evaluate and compare all of the possible feature subsets and select the one that yields the highest prediction accuracy. However, as the number of features increases, the number of possible subsets also increases according to a geometrical progression. For example, using a dataset with 1000 features, the number of all possible feature subsets is 2^1000^ ≈ 1.07 × 10^301^., which means that is virtually impossible to evaluate them in a reasonable time. Even if the problem space is reduced from 1000 to 100, the number of subsets for evaluation is 2^100^ ≈ 1.27 × 10^30^ cases, which will still require a long computational time. Therefore, it is practically impossible to calculate and compare all of the possible feature subsets because of the prohibitive computational cost.

Various approaches have been proposed to deal with feature selection from high dimensional datasets [2, 3], which can be divided into two general categories: the filter approach and feature subset selection. In the filter approach, each feature is evaluated using a specific evaluation measure, such as correlation, entropy, and consistency, to choose the best *n* features for further classification analysis. *Frequency-spatial domain decomposition (*FSDD) [4], Relief [5], chi-squared [6, 7], and gain ratio [8] are filter approaches. A feature selection algorithm based on a distance discriminant (FSDD) can identify features that allow good class separability among classes in each feature. The Relief algorithm randomly selects an instance and identifies its nearest neighbors, i.e., one from its own class and others from the other classes. The quality estimator is then updated for all of the attributes to assess how well the feature distinguishes the instance from its closest neighbors. Chi-squared is a well-known discrete data hypothesis testing method used in statistics, which evaluates the correlation between two variables and determines whether they are independent or correlated. The gain ratio is defined as the ratio between the information gain and the intrinsic value. The features with a higher gain ratio are selected.

Filter methods are effective in computational time, but they do not consider the interactions among the features. In particular, during gene expression data analysis, gene-gene interactions are an important issue that cannot be ignored. Feature subset selection is a better approach to this analysis [9] because it evaluates a set of features instead of each feature in a dataset. Therefore, the interactions among features can be measured in a natural manner using this approach. An important issue during feature subset selection is how to choose a reasonable number of subsets from all the subsets of features. Some heuristic methods have been proposed. Thus, forward search [10] starts from an empty set and sequentially adds the feature *x* that maximizes the evaluation value when combined with the previous feature subset that has already been selected. By contrast, backward elimination [10] starts from the full set and sequentially removes the feature *x* that least reduces the evaluation value. Hill climbing [10] starts with a random attribute set and evaluates all of its neighbors and chooses the best. Best first search [10] is similar to forward search but it also chooses the best node from those that have already been evaluated and it is then evaluated. The selection of the best node is repeated approximately *max.brackets* times if no better node is found. Minimum redundancy maximum relevance feature selection (MRMR) [11] combines forward search with redundancy evaluation.

Many feature (subset) selection methods have been proposed and applied to microarray analysis [12–15] and medical image analysis [16, 17]. Feature subset selection is a better approach for gene expression data than the filter approach, but it does not evaluate whole subsets of features because of the computational cost involved. Previous experimental results indicate that all pairs of two features can be evaluated within a reasonable time after appropriate preprocessing of all the features. Thus, if the interactions between pairs of two features are known, the interactions can be measured based on the classification accuracy for a given pair of features. Feature selection should be improved by applying this information in the filter method and feature subset selection approaches.

In the present study, we propose a method for improving the performance of feature selection algorithms using the pairwise classification accuracy results for two features by modifying previous feature selection algorithms. The results obtained in various experiments using microarray datasets confirmed that the proposed approach performance better than the original feature selection approach.

## Methods

Before describing the proposed approach, we need to define some notations. The input of feature selection is a dataset *DS*, which has *N* features and class labels *CL* for instances of features in *DS*. We denote *DS[i]* as the *i*-th feature in *DS*. The output of feature selection *CHOSEN* is the subset of features in *DS*. From a practical point of view, *CHOSEN* contains indexes of the selected features in *DS*. These notations are summarized as follows

- *DS*: input dataset, which has *N* features
- *DS[i]*: *DS[i]* for the *i*-th feature in *DS*
- *CL*: set of class labels for instances of features in *DS*
- *CHOSEN*: subset of selected features in *DS*

**Fig. 1:**
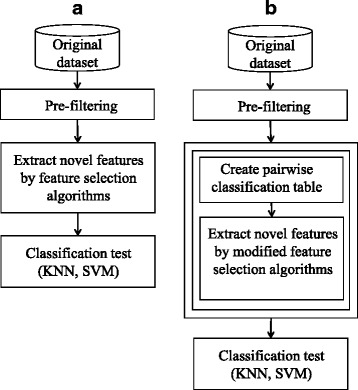
General (**a**) vs. proposed (**b**) feature selection processes

Figure 1a depicts the flow of the general feature selection process. The initial pre-filtering step removes highly irrelevant features according to feature evaluation and then extracts novel features by applying feature (subset) selection algorithms. The quality of the derived feature subset is evaluated by classification algorithms, such as k-nearest neighbor (KNN) and support vector machine (SVM). Figure 1b shows the flow of the proposed feature selection process. Our aim is to use evaluation information for the (*DS[i]*, *DS[j]*) pair. Evaluating the subsets of all features is impossible, but evaluating every (*DS[i]*, *DS[j]*) pair can be achieved within a reasonable amount of time. Including this information in the original feature selection should improve the quality of feature selection. The evaluation measure for (*DS[i]*, *DS[j]*) is not fixed and we use the classification accuracy as an evaluation measure in this study. We created a pairwise classification table, *COMBN*, and modified the original feature selection algorithms to use the *COMBN*.

**Fig. 2:**
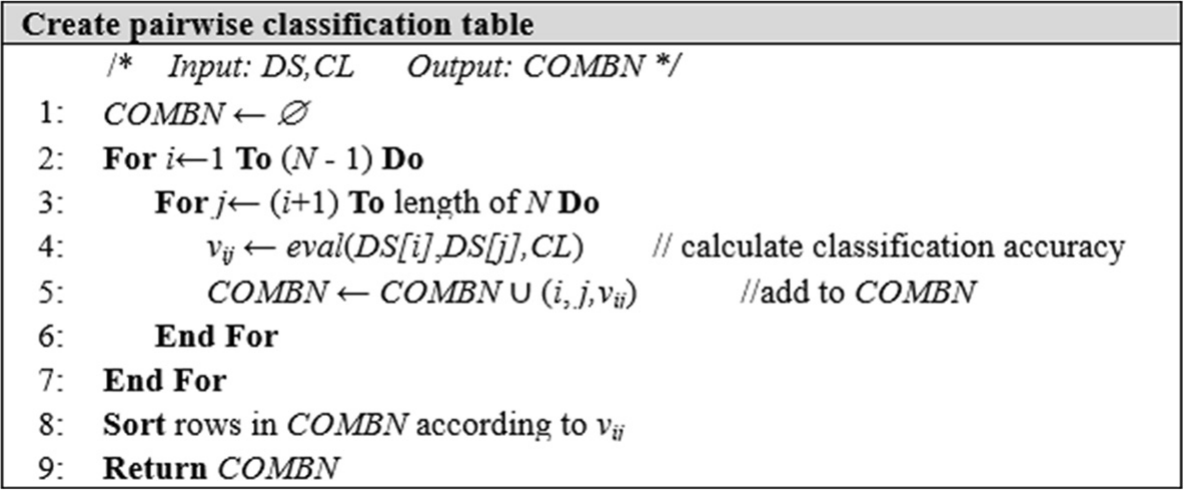
Algorithm of creating pairwise classification table

In the experiments, each dataset contained about 12000–15000 features. A mutual information test was performed for all of the features in a dataset and the best 1000 features were chosen in the pre-filtering step. In the proposed method, the input dataset *DS* for feature selection is this pre-filtered dataset. The *COMBN* pairwise classification table contains the (*i*, *j, v*_*ij*_) vector set, where *i*, *j* are the index of features *DS[i]*, *DS[j]* and *i ≠ j*, and *v*_*ij*_ is the classification accuracy for *DS[i]* and *DS[j]*. Various algorithms could be used to obtain the classification accuracy, but we employed a SVM. The length (number of rows) of the pairwise classification table is _1000_C_2_ = 499,500. Figure 2 describes the pseudo-code used to derive *COMBN*.

After producing *COMBN*, four filter algorithms, two feature subset selection algorithms, and MRMR are modified so the pairwise classification table is used in the original algorithms. Table 1 summarizes the modified feature selection algorithms.

**Table 1:** Feature selection algorithms modified according to the proposed approach

Group	Algorithm
Filter method	FSDD, Relief, Chi-squared, Gain ratio
Feature subset selection	Forward search, Backward elimination
Other	MRMR

**Fig. 3:**
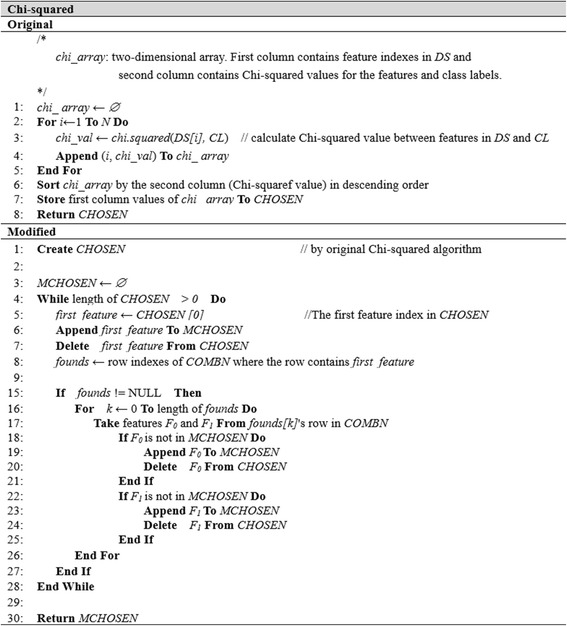
Algorithms of original and modified Chi-squared

**Fig. 4:**
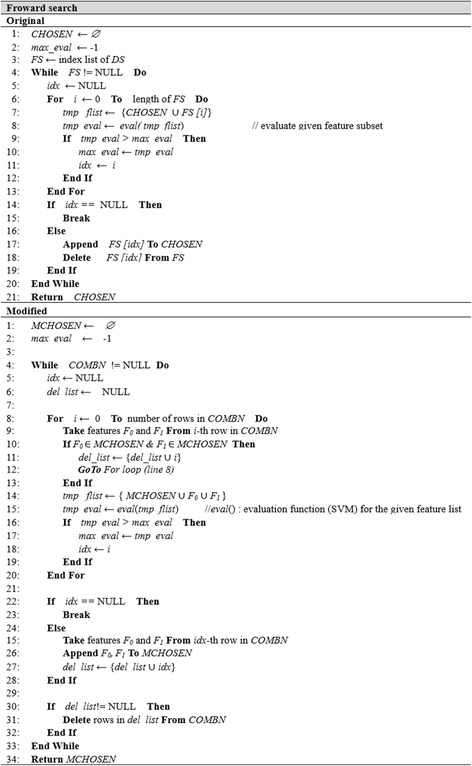
Algorithms of original and modified forward search

**Fig. 5:**
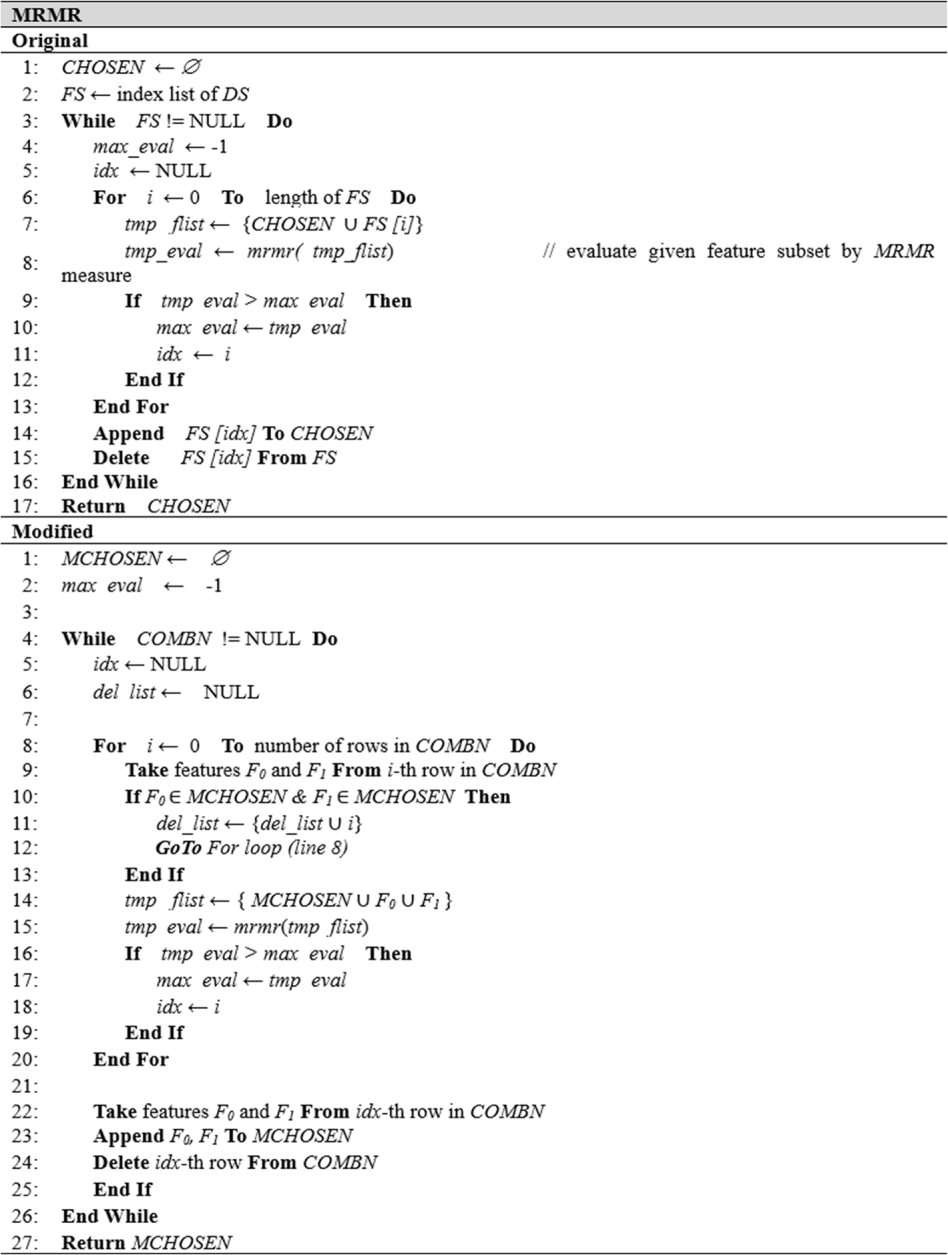
Algorithms of original and modified MRMR algorithms

The modification of the original feature selection algorithms is similar in most cases. Therefore, we present the pseudo-code for three selected algorithms, where Figs. 3, 4 and 5 show the pseudo-codes of the original and modified algorithms.

Figure 3 presents the Chi-squared pseudo-code as an example for the filter method. The original Chi-squared algorithm only calculates the Chi-squared value between each feature *DS[i]* and *CL*, and sorts the results in descending order. Finally, it returns the sorted list of feature indexes, *CHOSEN*. In the modified Chi-squared algorithm, we also use *CHOSEN* in the first step like the original method. We then pick the first feature index *first_feature* from *CHOSEN*, which is stored in *MCHOSEN* and removed from *CHOSEN (line 6,7)*. The next step is finding *first_feature* from *COMBN*. There may be multiple rows that match, so two features of matched rows are stored in *MCHOSEN* and removed from *CHOSEN* (*line 15–27*)*.* This process is repeated until *CHOSEN* is empty. As a result, the order of the feature index in *MCHOSEN* is different from that in *CHOSEN*. Users then select the first *M* features from *MCHOSEN* to use in the classification test. *MCHOSEN* is expected to obtain better accuracy than *CHOSEN*. The modified Chi-squared algorithm considers the Chi-squared evaluation value of each single feature and the interactions between pairs of features by referring to the pairwise classification information in *COMBN*.

The pseudo-codes of the original and modified forward search algorithm (Fig. 4) are used to modify the feature subset selection methods. The original forward search first algorithm finds a single feature with the highest evaluation value based on the *eval*() function and adds it to *CHOSEN*. In the second step, it repeatedly finds the next feature that can obtain the highest evaluation value together with the feature(s) in *CHOSEN* until no more features can increase the evaluation accuracy (*line 14,15*). Various methods are available for implementing the *eval*() function, but we employ SVM classification as an evaluation function. The modified algorithm finds the best two features from *COMBN* in the finding loop (*line 9*), whereas a single feature was searched from the feature list of *DS* in the original algorithm. This idea can be applied to other feature subset selection algorithms.

Figure 5 summarizes the pseudo-code for the original and modified MRMR algorithms. MRMR adopts the forward search method and evaluates the redundancy between target features, but there is no breaking condition for finding the feature subset. Therefore, it has characteristics of both the filter method and feature subset selection. Furthermore, MRMR uses mutual information for feature evaluation, so we need to convert the data values in *DS* into discrete values if the data values are continuous. The pseudo-code in Fig. 5 is similar to Fig. 4. However, the *eval()* function in Fig. 4 is substituted by the *mrmr()* function and breaking conditions in Fig. 4 are omitted (*see line 14,15 for original forward search*).

After obtaining the selected feature subsets produced by several algorithms, a classification test was performed using SVM and KNN because they are recognized for their good performance. The leave-one-out cross-validation test was used to avoid the overfitting problem. The FSelector package [18] in R (http://www.r-project.org) was used to test the original feature selection algorithms. FSDD and MRMR are not supported by the FSelector package, so they were implemented using R.

## Results

To compare the original and proposed feature selection algorithms, we used five microarray datasets from the Gene Expression Omnibus (GEO) website (http://www.ncbi.nlm.nih.gov/geo/), which provides accession IDs for GEO datasets. A brief description of the datasets is provided in Table 2.

**Table 2:** Descriptions of the datasets

No.	Dataset Name	Number of features	Number of samples	Number of classes
1	GDS1027	15897	154	4
2	GDS2545	12558	171	4
3	GDS2546	12553	167	4
4	GDS2547	12579	164	4
5	GDS3715	12626	109	3

**Fig. 6:**
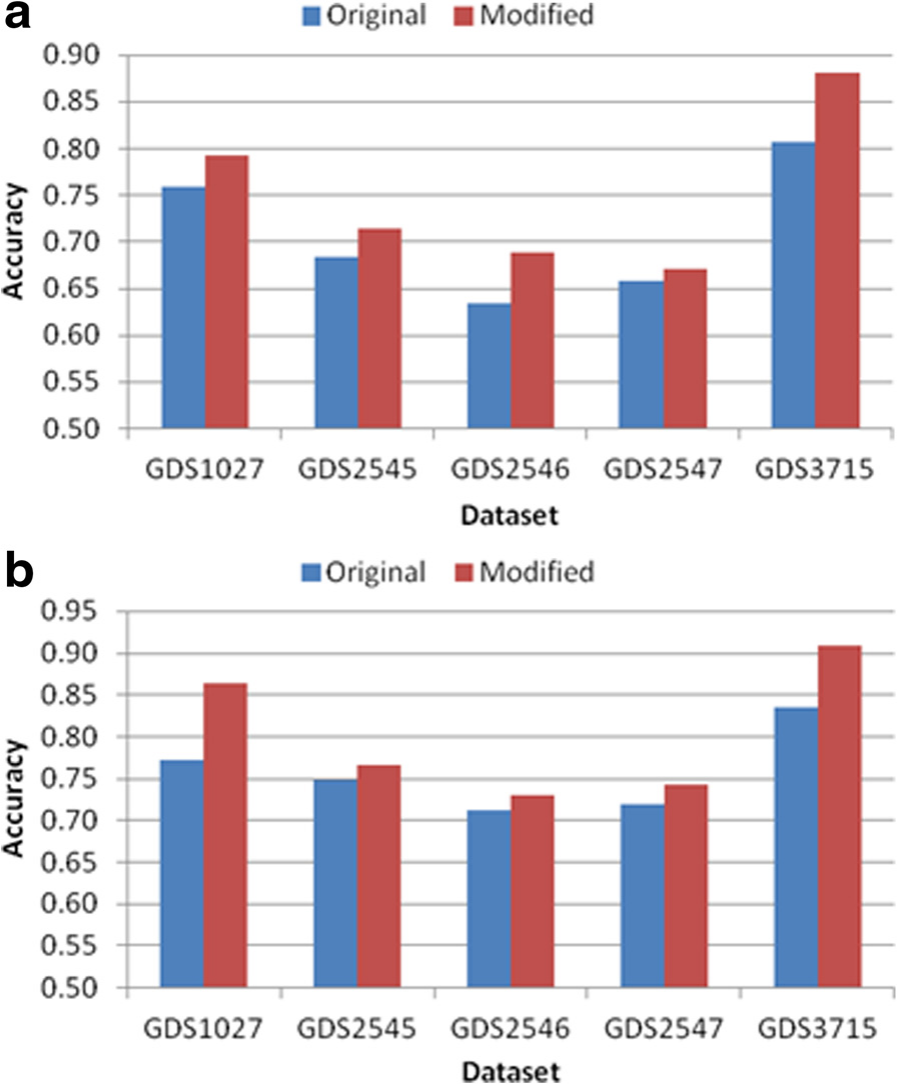
Comparison of maximum classification accuracy between original MRMR and proposed method. **a** KNN classification **b** SVM classification

**Fig. 7:**
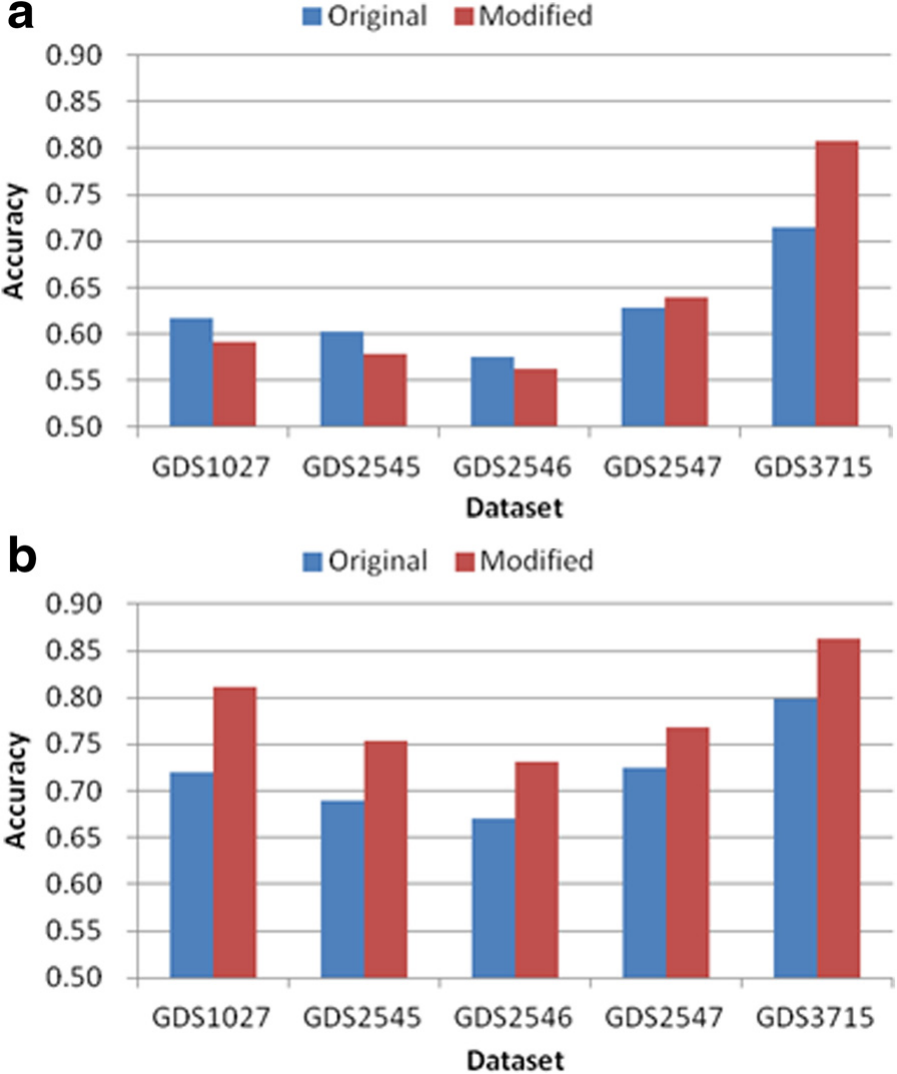
Comparison of maximum classification accuracy between original FSDD and proposed method. **a** KNN classification **b** SVM classification

**Fig. 8:**
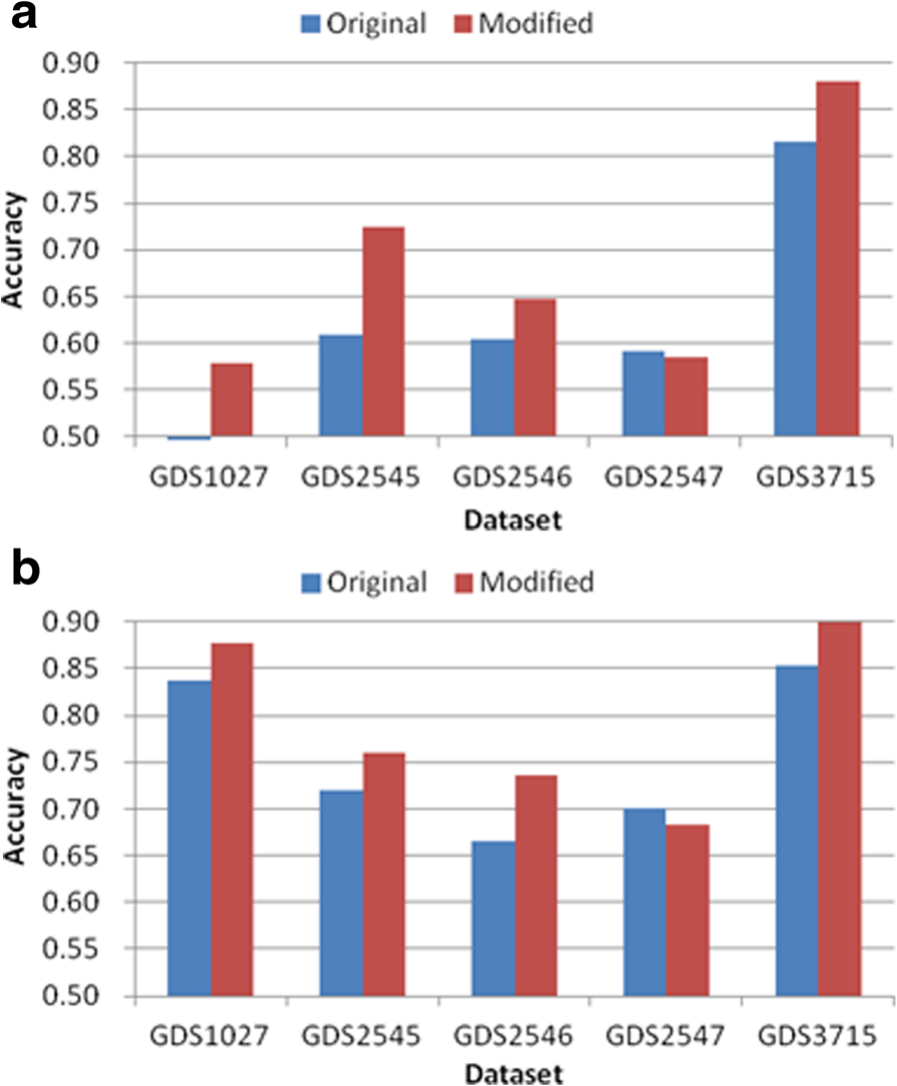
Comparison of maximum classification accuracy between original Relief and proposed method. **a** KNN classification **b** SVM classification

**Fig. 9:**
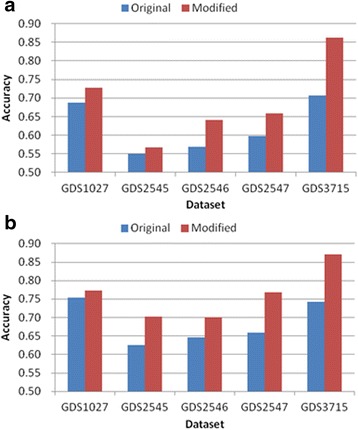
Comparison of maximum classification accuracy between original Chi-squared and proposed method. **a** KNN classification **b** SVM classification

**Fig. 10:**
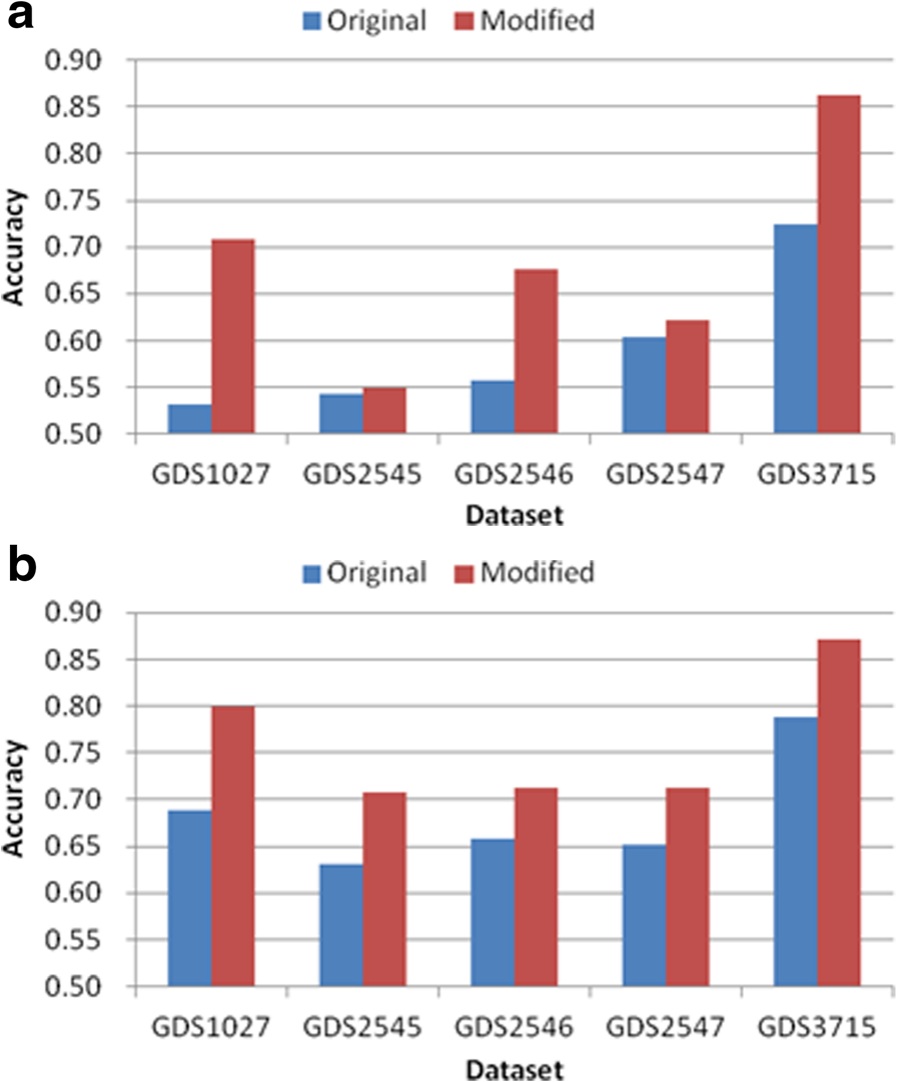
Comparison of maximum classification accuracy between original Gain ratio and proposed method. **a** KNN classification **b** SVM classification

Tables 3, 4, 5, 6 and 7 and Figs. 6, 7, 8, 9 and 10 show the experimental results obtained by the filter methods and MRMR to compare the classification accuracy of the original feature selection algorithms and proposed methods. The filter methods evaluate each feature and the user must select the best *n* features from the evaluation results. For most of the datasets and with various numbers of selected features, the proposed modified algorithms obtained higher classification accuracy than the original methods. In some cases for FSDD and Relief, the original algorithms were marginally more accurate than the proposed methods with the KNN test. The SVM test always improved the classification accuracy, excluding one result obtained by Relief. In general, the SVM yielded greater improvements than KNN, possibly because the pairwise classification table was produced by the SVM, and thus the KNN test might have made greater improvements if it was used instead. In general, the proposed method increased the classification accuracy by 2–11 % and it was most accurate when the number of features selected was 25.

**Table 3:** Comparison of the classification accuracy using the original MRMR and the proposed method

MRMR		GDS1027		GDS2545		GDS2546		GDS2547		GDS3715	
		Orig	Modi	Orig	Modi	Orig	Modi	Orig	Modi	Orig	Modi
5	KNN	0.73	0.69	**0.68**	0.62	0.60	**0.69**	0.57	0.66	0.78	0.84
	SVM	0.76	0.73	0.73	0.71	0.68	0.68	0.70	**0.74**	0.83	0.85
10	KNN	0.70	0.71	0.56	0.63	**0.63**	0.60	0.63	**0.67**	0.73	0.85
	SVM	0.70	0.75	0.73	0.76	**0.71**	0.68	0.67	0.73	0.77	0.89
15	KNN	0.71	0.75	0.60	0.61	0.59	0.60	0.63	0.54	0.79	0.84
	SVM	0.75	0.80	0.72	0.75	0.70	**0.73**	0.71	0.73	0.79	0.87
20	KNN	**0.76**	0.75	0.63	**0.71**	0.60	0.62	0.65	0.62	**0.81**	0.84
	SVM	**0.77**	0.81	0.71	0.75	0.65	0.69	0.70	0.71	0.82	0.90
25	KNN	0.75	**0.79**	0.61	0.67	0.63	0.62	**0.66**	0.60	0.81	0.86
	SVM	0.77	**0.86**	0.71	**0.77**	0.69	0.69	**0.72**	0.73	**0.83**	0.90
30	KNN	0.75	0.77	0.63	0.63	0.60	0.66	0.63	0.59	0.80	**0.88**
	SVM	0.77	0.86	**0.75**	0.77	0.69	0.67	0.72	0.73	0.83	**0.91**
MAX KNN		0.76	0.79	0.68	0.71	0.63	0.69	0.66	0.67	0.81	0.88
MAX SVM		0.77	0.86	0.75	0.77	0.71	0.73	0.72	0.74	0.83	0.91

*Orig* Original algorithm, *Modi* Proposed modified algorithm

Values in the first column are presented as the number of features selected for the classification test and the others are presented as classification accuracy. The bold numbers denote the highest value of KNN and SVM of each column

**Table 4:** Comparison of the classification accuracy using the original FSDD and the proposed method

FSDD		GDS1027		GDS2545		GDS2546		GDS2547		GDS3715	
		Orig	Modi	Orig	Modi	Orig	Modi	Orig	Modi	Orig	Modi
5	KNN	0.53	0.58	0.44	0.52	0.52	0.45	0.52	0.54	0.57	0.73
	SVM	0.58	0.68	0.59	0.64	0.59	0.66	0.58	0.68	0.71	0.79
10	KNN	0.55	**0.59**	0.51	0.49	0.53	0.53	**0.63**	0.54	0.68	0.78
	SVM	0.65	0.71	0.64	**0.75**	0.59	0.65	0.64	0.73	0.71	**0.86**
15	KNN	0.56	0.59	0.51	0.48	0.54	0.54	0.60	0.59	0.70	0.75
	SVM	0.66	0.73	0.67	0.71	0.63	0.68	0.66	**0.74**	0.72	0.84
20	KNN	0.56	0.57	0.54	0.54	**0.57**	0.55	0.61	**0.64**	0.66	0.76
	SVM	0.70	0.77	0.68	0.74	0.66	0.70	0.70	0.77	0.72	0.83
25	KNN	**0.62**	0.59	**0.60**	**0.58**	0.57	0.55	0.60	0.59	**0.72**	0.79
	SVM	**0.72**	0.77	0.68	0.74	**0.67**	**0.73**	0.70	0.76	0.79	0.85
30	KNN	0.60	0.58	0.60	0.57	0.54	**0.56**	0.62	0.60	0.71	**0.81**
	SVM	0.69	**0.81**	**0.69**	0.74	0.65	0.72	**0.73**	0.72	**0.80**	0.85
MAX KNN		0.62	0.59	0.60	0.58	0.57	0.56	0.63	0.64	0.72	0.81
MAX SVM		0.72	0.81	0.69	0.75	0.67	0.73	0.73	0.77	0.80	0.86

*Orig* Original algorithm, *Modi* Proposed modified algorithm

Values in the first column are presented as the number of features selected for the classification test and the others are presented as classification accuracy. The bold numbers denote the highest value of KNN and SVM of each column

**Table 5:** Comparison of the classification accuracy using the original Relief and the proposed method

Relief		GDS1027		GDS2545		GDS2546		GDS2547		GDS3715	
		Orig	Modi	Orig	Modi	Orig	Modi	Orig	Modi	Orig	Modi
5	KNN	0.39	0.52	0.59	0.65	0.56	0.56	0.45	0.59	0.71	0.72
	SVM	0.46	0.71	0.65	0.72	0.62	0.62	0.55	0.67	0.77	0.83
10	KNN	0.45	0.51	0.59	0.69	0.54	0.56	0.43	0.57	0.72	0.80
	SVM	0.66	0.73	**0.72**	0.72	**0.66**	0.71	0.59	0.62	0.81	0.82
15	KNN	**0.47**	0.53	0.57	0.71	0.59	0.57	0.46	0.58	**0.82**	0.84
	SVM	0.76	0.77	0.71	0.74	0.62	**0.74**	0.60	0.62	0.83	0.84
20	KNN	0.47	0.55	**0.61**	0.70	0.56	**0.65**	0.51	0.57	0.76	0.84
	SVM	0.81	0.80	0.71	0.74	0.66	0.74	0.66	0.68	0.82	0.86
25	KNN	0.44	0.54	0.56	0.70	0.55	0.60	0.57	**0.59**	0.73	**0.88**
	SVM	0.82	0.87	0.71	**0.76**	0.66	0.72	0.68	**0.68**	0.83	**0.90**
30	KNN	0.45	**0.58**	0.56	**0.73**	**0.60**	0.60	**0.59**	0.57	0.78	0.86
	SVM	**0.84**	**0.88**	0.70	0.76	0.66	0.70	**0.70**	0.68	**0.85**	0.88
MAX KNN		0.47	0.58	0.61	0.73	0.60	0.65	0.59	0.59	0.82	0.88
MAX SVM		0.84	0.88	0.72	0.76	0.66	0.74	0.70	0.68	0.85	0.90

*Orig* Original algorithm, *Modi* Proposed modified algorithm

Values in the first column are presented as the number of features selected for the classification test and the others are presented as classification accuracy. The bold numbers denote the highest value of KNN and SVM of each column

**Table 6:** Comparison of the classification accuracy using the original Chi-squared and the proposed method

Chi Squared		GDS1027		GDS2545		GDS2546		GDS2547		GDS3715	
		Orig	Modi	Orig	Modi	Orig	Modi	Orig	Modi	Orig	Modi
5	KNN	0.51	0.68	0.47	0.54	0.44	0.59	0.51	0.55	0.61	0.73
	SVM	0.61	0.69	0.60	0.67	0.51	0.69	0.58	0.65	0.72	0.82
10	KNN	0.60	0.64	0.49	**0.57**	0.50	0.57	0.51	0.58	0.70	0.78
	SVM	0.65	0.74	0.61	0.67	0.57	0.66	0.62	0.70	0.69	0.87
15	KNN	0.64	0.66	0.47	0.53	0.55	0.63	0.49	0.60	0.67	0.75
	SVM	0.69	0.73	0.60	0.67	**0.65**	0.66	0.60	0.70	0.73	0.85
20	KNN	0.64	0.70	0.51	0.53	0.54	**0.64**	0.56	0.64	0.67	0.83
	SVM	0.73	**0.77**	0.60	**0.70**	0.63	0.69	0.65	0.74	0.72	0.86
25	KNN	**0.69**	**0.73**	0.53	0.52	**0.57**	0.61	0.59	**0.66**	0.67	**0.86**
	SVM	0.73	0.77	**0.63**	0.68	0.63	**0.70**	**0.66**	**0.77**	0.70	0.85
30	KNN	0.62	0.71	**0.55**	0.53	0.54	0.60	**0.60**	0.65	**0.71**	0.85
	SVM	**0.75**	0.77	0.61	0.68	0.63	0.70	0.66	0.74	**0.74**	**0.87**
MAX KNN		0.69	0.73	0.55	0.57	0.57	0.64	0.60	0.66	0.71	0.86
MAX SVM		0.75	0.77	0.63	0.70	0.65	0.70	0.66	0.77	0.74	0.87

*Orig* Original algorithm, *Modi* Proposed modified algorithm

Values in the first column are presented as the number of features selected for the classification test and the others are presented as classification accuracy. The bold numbers denote the highest value of KNN and SVM of each column

**Table 7:** Comparison of the classification accuracy using the original Gain ratio and the proposed method

Gain Ratio		GDS1027		GDS2545		GDS2546		GDS2547		GDS3715	
		Orig	Modi	Orig	Modi	Orig	Modi	Orig	Modi	Orig	Modi
5	KNN	0.36	0.60	0.49	0.51	0.44	0.55	0.53	0.55	0.58	0.76
	SVM	0.53	0.75	0.60	0.61	0.51	0.62	0.60	0.65	0.66	0.82
10	KNN	0.37	0.62	0.47	0.53	0.53	0.57	0.51	0.60	0.69	0.79
	SVM	0.60	0.71	0.60	0.64	0.56	0.66	0.59	**0.71**	0.73	0.86
15	KNN	0.47	0.64	0.50	0.55	0.53	0.62	0.50	**0.62**	0.72	0.82
	SVM	0.65	0.67	0.60	0.65	0.63	0.66	0.59	0.68	0.77	**0.87**
20	KNN	0.42	0.66	0.52	0.53	0.55	0.65	0.52	0.62	**0.72**	0.82
	SVM	**0.69**	0.79	0.62	0.67	0.64	0.68	0.62	0.70	**0.79**	0.86
25	KNN	**0.53**	**0.71**	0.51	0.54	**0.56**	**0.68**	**0.60**	0.62	0.69	0.82
	SVM	0.67	**0.80**	**0.63**	0.67	**0.66**	**0.71**	**0.65**	0.70	0.79	0.87
30	KNN	0.53	0.68	**0.54**	**0.55**	0.56	0.64	0.60	0.62	0.65	**0.86**
	SVM	0.68	0.77	0.61	**0.71**	0.66	0.70	0.65	0.69	0.79	0.86
MAX KNN		0.53	0.71	0.54	0.55	0.56	0.68	0.60	0.62	0.72	0.86
MAX SVM		0.69	0.80	0.63	0.71	0.66	0.71	0.65	0.71	0.79	0.87

*Orig* Original algorithm, *Modi* Proposed modified algorithm

Values in the first column are presented as the number of features selected for the classification test and the others are presented as classification accuracy. The bold numbers denote the highest value of KNN and SVM of each column

**Fig. 11:**
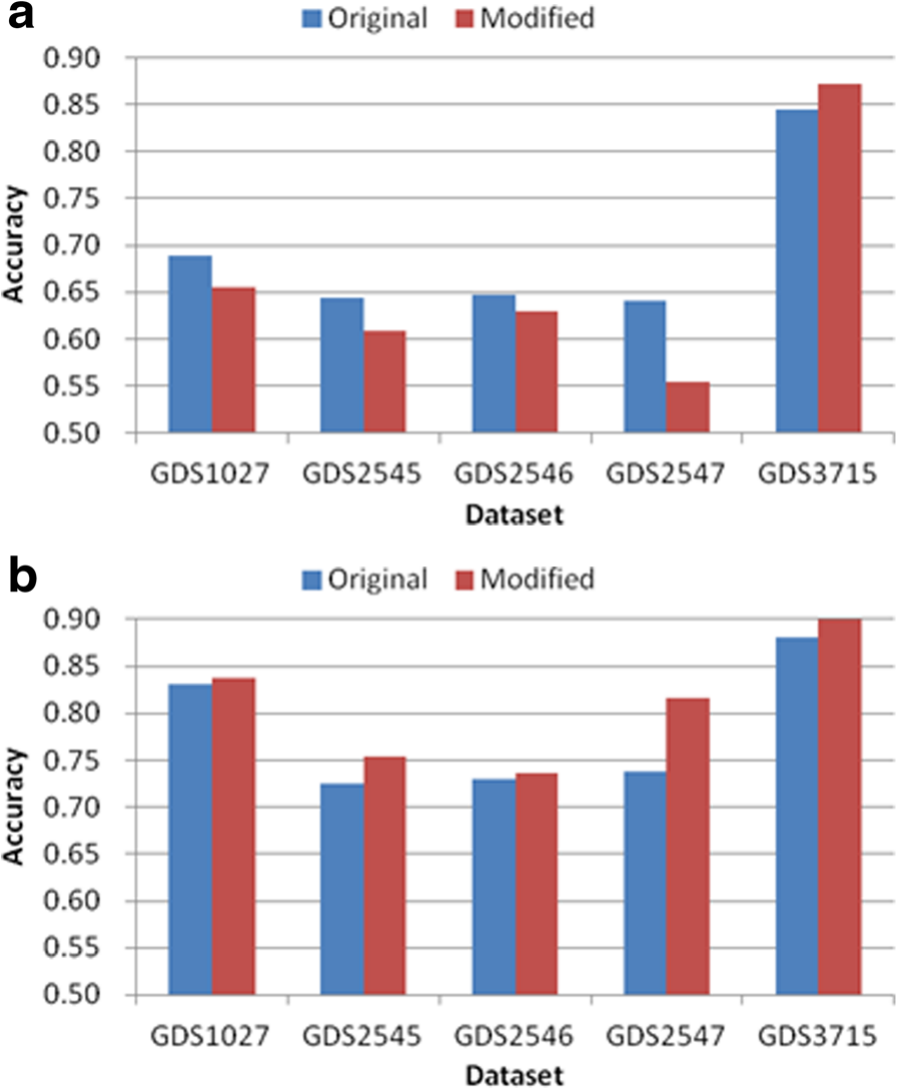
Comparison of maximum classification accuracy between original forward search and proposed method. **a** KNN classification **b** SVM classification

**Fig 12:**
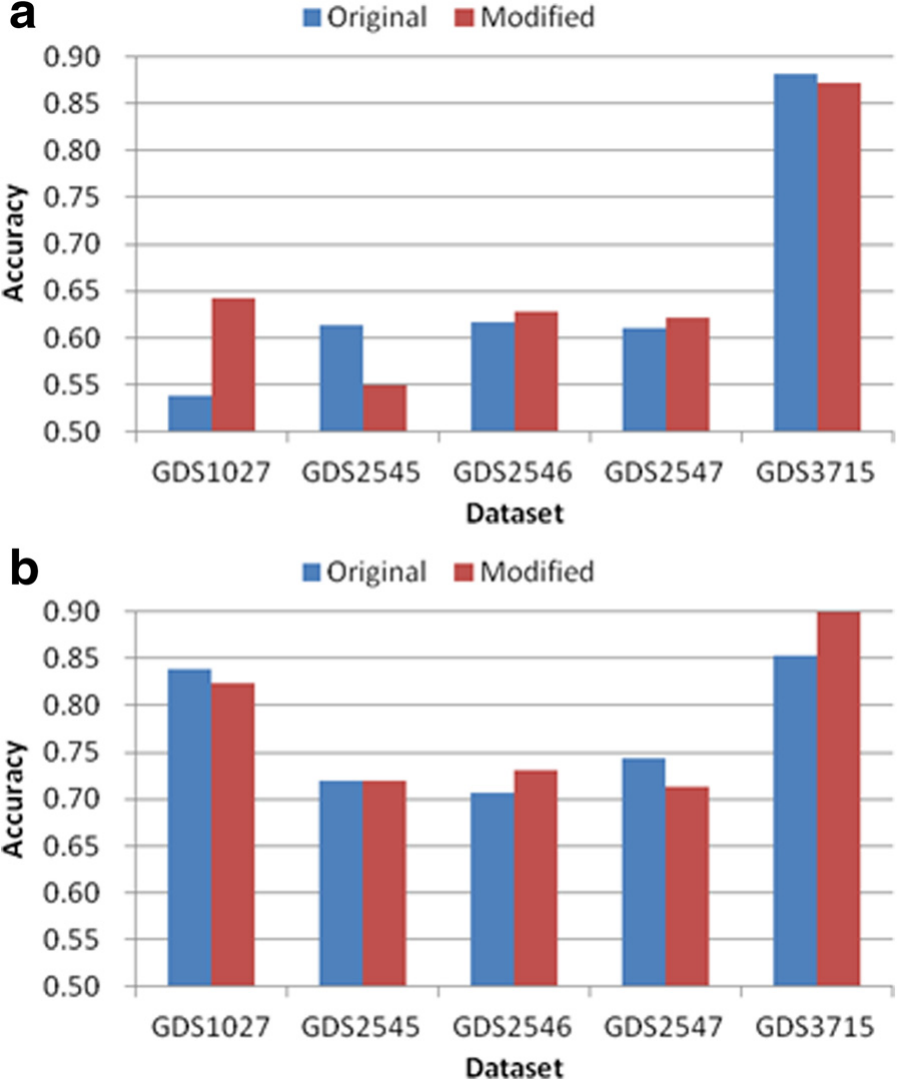
Comparison of maximum classification accuracy between original backward elimination and proposed method. **a** KNN classification **b** SVM classification

Tables 8 and 9, and Figs. 11 and 12 show the experimental results obtained by the feature subset selection algorithms. In the case of forward search (Table 8 and Fig. 11), the SVM test obtained a marginal improvement in the classification accuracy compared with the original method, whereas the KNN test decreased the accuracy. The difference between KNN and SVM may have been due to the method employed for the preparation of the pairwise classification table. Thus, if the *eval()* function in Figs. 2 and 4 had been changed to KNN, the results in Fig. 11(a) would be different. The proposed method markedly improved the accuracy of the filter methods compared with feature subset selection. The filter methods only evaluate each feature and they do not consider interactions between features, whereas feature subset selection methods consider feature interactions. Therefore, the proposed method performed well with the filter methods. The proposed method selected features with greater numbers than the original algorithms and improved the classification accuracy (Table 8). In the case of forward search (Table 9 and Fig. 12), the original algorithm did not reduce the number of features, whereas the proposed method reduced the initial 1000 features by 90 %. The proposed method removed a large number of features, but the KNN and SVM tests improved the classification accuracy. Thus, the proposed method has greater selective power than the original.

**Table 8:** Comparison of the classification accuracy using the original forward search and the proposed method

Forward	GDS1027		GDS2545		GDS2546		GDS2547		GDS3715	
	Orig	Modi	Orig	Modi	Orig	Modi	Orig	Modi	Orig	Modi
KNN	0.69	0.66	0.64	0.61	0.65	0.63	0.64	0.55	0.84	0.87
SVM	0.83	0.84	0.73	0.75	0.73	0.749	0.74	0.82	0.88	0.91
Ft number	11	14	10	15	8	10	9	16	8	13

*Orig* Original algorithm, *Modi* Proposed modified algorithm

**Table 9:** Comparison of the classification accuracy using the original backward elimination and the proposed method

Backward	GDS1027		GDS2545		GDS2546		GDS2547		GDS3715	
	Orig	Modi	Orig	Modi	Orig	Modi	Orig	Modi	Orig	Modi
KNN	0.54	0.64	0.61	0.55	0.62	0.63	0.61	0.62	0.84	0.87
SVM	0.84	0.82	0.72	0.72	0.71	0.73	0.74	0.71	0.85	0.91
Ft number	1000	123	1000	92	1000	174	1000	121	1000	34

*Orig* Original algorithm, *Modi* Proposed modified algorithm

To evaluate the execution time for the proposed method, we tested the execution time using a personal computer equipped with an Intel(R) Core(TM) i5-4690 @ 3.5 GHz CPU, 16 GByte main memory, and the Windows 8.1 operating system. The proposed method requires an extra step for the pairwise classification accuracy table and Table 10 summarizes the computational time needed for this step. The average time was 63.1 min. This step is performed only once for given datasets and it is not a great burden for the overall feature selection process. Table 11 summarizes the computational time required by various algorithms using the GDS1027 dataset. The proposed modified algorithms were faster than the original algorithms in the case of Relief, forward search, and MRMR, but slower for FSDD and Chi-squared. In general, the proposed algorithms produced the results within a reasonable amount of time.

**Table 10:** Execution time required to create the pairwise classification table for each dataset

Dataset	Execution time, minutes
GDS1027	63.7
GDS2545	74.3
GDS2546	71.5
GDS2547	68.6
GDS3715	37.4

**Table 11:** Comparison of the execution times (minutes) for the original and modified algorithms

Algorithm	Original	Proposed
MRMR	6.81	4.5
FSDD	0.01	1.3
Relief	3.09	0.08
Chi-squared	0.05	0.07
Forward search	1.39	1.38

## Discussion

The proposed algorithms are useful but their implementation may be a difficult task for users. Thus, to facilitate further research, we have built an R package called “fsPair” and posted it on the web site (http://bitl.dankook.ac.kr/biosw/pairwise). This package includes executable codes, source codes, a user manual, usage examples, and a sample dataset. We have added three more classifiers, i.e., random forest, naive Bayes, and neural network. We have also added multi-core parallelism to allow the rapid generation of pairwise classification tables. Users are free to download this package and test the proposed feature selection methods using their own datasets.

**Fig. 13:**
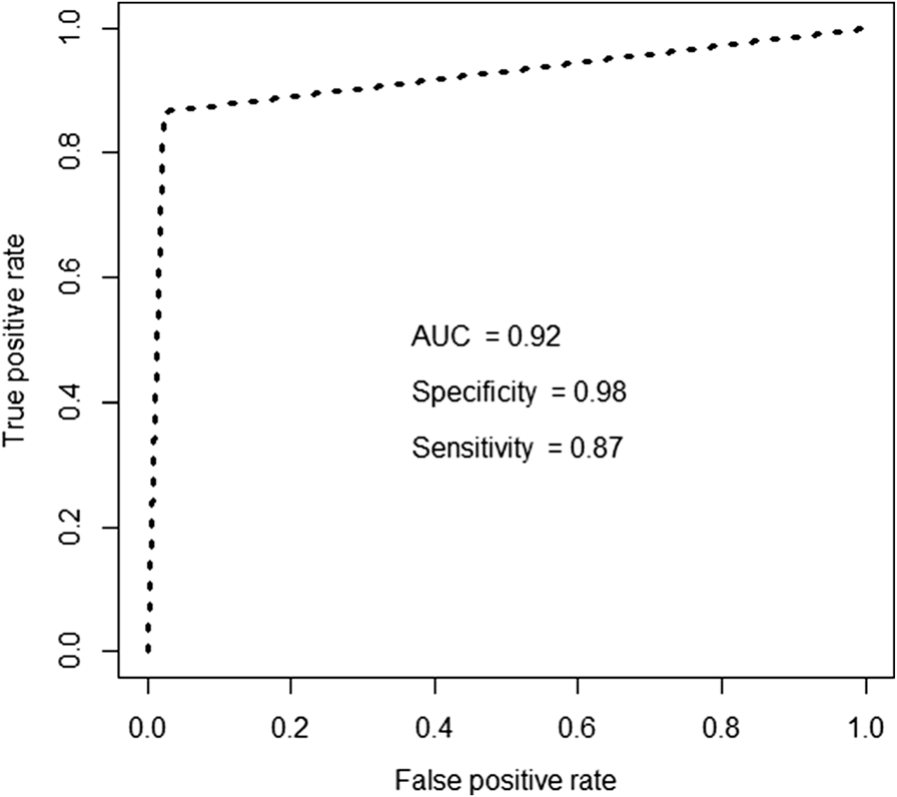
ROC analysis for new dataset that has five selected features

Next, we consider the application of the proposed methods to the solution of real problems. Kurgan et al. [19] proposed a method for cardiac diagnosis using single proton emission computed tomography (SPECT) images, where they built the SPECTF dataset containing 44 features and 267 instances. Each of the features contained values extracted from a specific region of interest. Each of the patients (instances) was classified according to two categories: normal and abnormal. They aimed to produce a good classifier for diagnosing the problem. The accuracy of their proposed CLIP3 algorithm was 77 %. We tried to find “marker features” that might be helpful for cardiac diagnosis. Thus, using our fsPair package and the original algorithms, we test different combinations of feature selection algorithms and classifiers, and Table 12 summarizes the results obtained. Using the SPECTF dataset, the results produced by the original and modified algorithms differed little because the dataset had a small number of features. However, the proposed algorithms selected a smaller numbers of features than the original algorithms, but their accuracy was similar. For example, the original algorithms had the best accuracy using MRMR and random forest with 15 features, whereas the modified algorithms had the best accuracy using FSDD and random forest with five features. Thus, five features referred to as F21S, F17R, F20S, F3S, F13S, and F8S are highly informative features for cardiac diagnosis. We performed a bootstrap test using the five features from the dataset and a very good area under the receiver operating characteristic curve (AUC) score was obtained, as shown in Fig. 13. This suggests that the five features selected may be of practical value for future diagnosis.

**Table 12:** Classification accuracy and number of features selected with five classifiers and six feature selection algorithms

		Forward	Backward	Relief	FSDD	Chi-squared	MRMR
KNN	Original	0.781/01	0.785/41	0.716/10	0.776/05	0.795/05	0.767/15
	Modified	0.775/10	0.755/23	0.713/05	0.793/15	0.770/10	0.748/15
SVM	Original	0.801/01	0.796/41	0.799/10	0.805/15	0.797/05	0.800/10
	Modified	0.812/11	0.820/23	0.798/10	0.823/15	0.802/30	0.809/20
NB	Original	0.793/01	0.777/41	0.790/20	0.776/05	0.763/05	0.805/15
	Modified	0.819/12	0.754/23	0.813/05	0.813/05	0.788/10	0.786/20
RF	Original	0.783/01	0.791/41	0.808/30	0.806/20	0.825/15	**0.830/15**
	Modified	0.843/12	0.820/23	0.823/30	**0.851/05**	0.801/15	0.819/20
NN	Original	0.795/01	NA	0.771/30	0.791/05	0.771/15	0.782/10
	Modified	0.744/11	0.734/23	0.759/25	0.764/15	0.765/05	0.771/30

(*NB* Naive Bayes, *RF* Random Forest, *NN* Neural Network)

## Conclusions

Feature (subset) selection has various applications in bioinformatics. However, the selection of a novel feature set from a huge numbers of features is a critical issue, which involves the evaluation of each feature, feature interaction, and redundancy in the features. In this study, we proposed a method that improves the quality of feature selection. Using information about the interactions between two features is very helpful for enhancing the original feature selection algorithms. If the computational power increases in the future, then information about the interactions between three or more features in a given dataset could further improve the feature selection process. The generation of interaction information is another issue. In this study, we used the classification accuracy as an evaluation measure for interaction but the evaluation measure could be changed if the aim of feature selection is not classification. The proposed method does not include redundancy among its features. Thus, the addition of a redundancy removal algorithm may yield better results and this will be explored in future research.

## Abbreviations

FSDD: *Frequency-spatial domain decomposition*
GEO: Gene expression omnibus
KNN: K-nearest neighbor
*MRMR*: Minimum redundancy maximum relevance
SVM: Support vector machine
